# Symmetry Breaking around Aqueous Ammonia Revealed in Nitrogen K-edge X-ray Absorption

**DOI:** 10.1021/acs.jpclett.4c03625

**Published:** 2025-03-27

**Authors:** Michael Odelius, Sarai Dery Folkestad, Thanit Saisopa, Yuttakarn Rattanachai, Wutthigrai Sailuam, Hayato Yuzawa, Nobuhiro Kosugi, Alexander C. Paul, Henrik Koch, Denis Céolin

**Affiliations:** † Department of Physics, Stockholm University, 10691 Stockholm, Sweden; ‡ Department of Chemistry, Norwegian University of Science and Technology, NTNU, 7491 Trondheim, Norway; ¶ Department of Applied Physics, Faculty of Sciences and Liberal Arts, Rajamangala University of Technology Isan, Nakhon Ratchasima 30000, Thailand; § Department of Applied Physics, Faculty of Engineering, Rajamangala University of Technology ISAN (Khon Kaen Campus), Khon Kaen 40000, Thailand; ∥ UVSOR Synchrotron Facility, Institute for Molecular Science, Okazaki 444-8585, Japan; ⊥ Synchrotron SOLEIL, L’Orme des Merisiers, BP 48, St Aubin, 91192 Gif sur Yvette, France

## Abstract

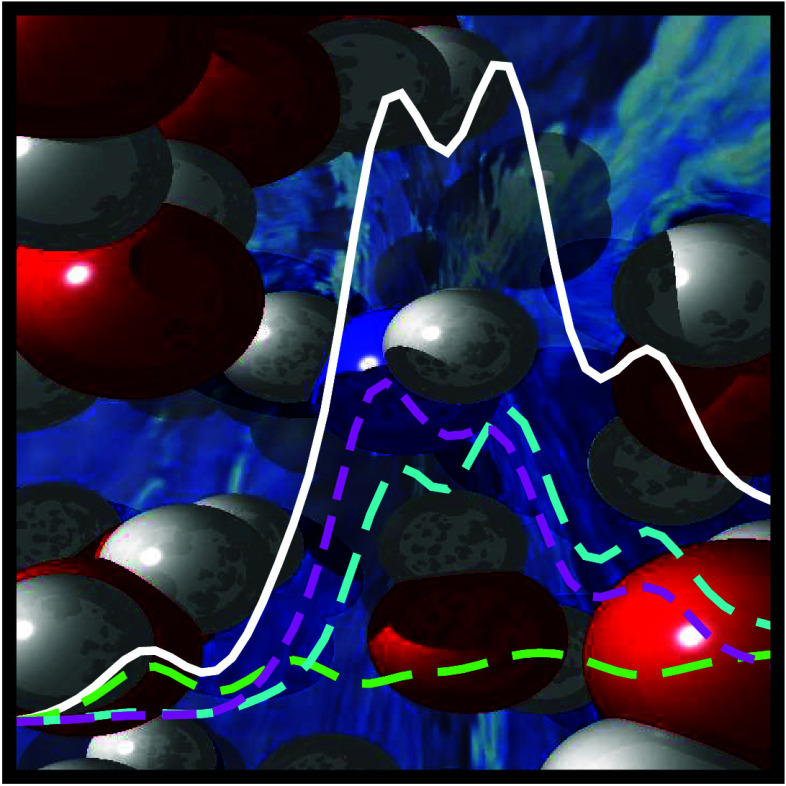

Nitrogen K-edge X-ray absorption (XA) spectroscopy of aqueous ammonia reveals a splitting in the main-edge, which through theoretical modeling is shown to be related to symmetry breaking in hydrogen bonding. The XA main-edge of NH_3_ is formed by a pair of degenerate core-excitations into extended molecular orbitals. In aqueous solution, these form an antibonding mixture with orbitals of the surrounding water molecules. Although the spectral response to distortions is complex, we show that the degeneracy of the core-excitations is lifted by asymmetry in hydrogen bond donation (NH···O). A quantitative relation between asymmetry in the hydration shell and splitting in the main-edge of the nitrogen K-edge XA spectrum is established from systematic symmetry breaking in well-defined cluster models and through molecular dynamics sampling of simulated XA spectra of aqueous ammonia. The finding indicates that XA spectroscopy is a sensitive probe of asymmetry in solvation also around functional groups in biomolecules.

Detailed molecular scale insight into hydration and fluctuations of the hydrogen bond network is important for a fundamental understanding of aqueous solutions and for applications in many fields, including biochemistry and inorganic chemistry. Neutron and X-ray scattering experiments and molecular dynamics simulations have proven to be a powerful combination to study the structure and dynamics of aqueous solutions^1−4^ yielding a deep understanding of solvation. Local vibrational Raman^5^ and infrared^6,7^ probes give information on hydrogen bond strength and dynamics in solvation shells and the hydrogen bond network. More recently, also the electronic probes of X-ray photoelectron spectroscopy,^8^ X-ray absorption (XA) spectroscopy^9,10^ as well as resonant and nonresonant X-ray emission spectroscopy^11,12^ have enabled studies of the electronic structure and chemical bonding in liquid water and aqueous solutions. In a broader photon energy range, extended X-ray absorption fine structure (EXAFS) can give information on the coordination of heavy elements at short and long distances.

The strengths of hydrogen bonding in aqueous ammonia and ammonium have been probed in infrared spectroscopy^13,14^ and Raman spectroscopy^15,16^ complemented by structural information from X-ray scattering.^15^ Probing how the electronic structure is influenced by solvent fluctuations is important for understanding chemical bonding and reactivity. X-ray absorption is an element-specific local probe sensitive to both structural and electronic degrees of freedom. XA spectroscopy is very sensitive to the chemical environment, since it, in the language of molecular orbital theory, is probing extended unoccupied orbitals of the excited molecule mixing with orbitals of the surroundings. X-ray photons possess enough energy to excite inner electrons, located near the atom cores, and the photon energy can be tuned to target specific core-levels; the nitrogen 1s orbital in the case of aqueous ammonia. Nitrogen K-edge XA spectra have been recorded previously for aqueous ammonia NH_3_(aq) in fluorescence and transmission mode^13,17^ and for the related systems of ammonium, ethylammonium ions (Et_*x*_NH_4–x_^+^, x = 1,2,3,4) and amino acids.^13,18,19^ Soft X-rays excite the nitrogen 1s core–electron to unoccupied orbitals according to their local (2p,3p,···) character which is influenced by ionic interactions and hydration, specifically hydrogen bonding. Ammonia has also been studied with X-ray photoelectron spectroscopy.^20,21^ X-ray induced ultrafast charge dynamics in aqueous ammonia has been probed through Auger-Meitner decay.^22,23^ Chemical bonding and ultrafast nuclear dynamics have been investigated with resonant inelastic X-ray scattering.^24^

X-ray spectroscopy requires theoretical modeling for an accurate interpretation of the measured data, and many different approaches are available for simulating the spectra. We have previously employed ab initio molecular dynamics (AIMD) simulations, based on classical forces from density functional theory (DFT), and the transition potential (TP-)DFT spectrum simulations, in the excited full core-hole (XFH) approximation.^25^ DFT calculations have also been employed in combined experimental and theoretical studies of X-ray emission spectra of aqueous ammonia.^17^ The TP-DFT method gives an approximate molecular orbital representation of the X-ray spectra, but has the advantage of allowing for simulations in extended (periodic) models of the solutions. Complementarily, accurate coupled cluster calculations^26−28^ with a proper state representation of the core-excitations have been developed, but these are restricted to cluster models based on cutouts from the extended simulation trajectories. The level of coupled cluster calculations (CC3 versus CCSD) and the selection of the active region in the multilayer with embedding in Hartree–Fock (MLCC3-in-HF) calculations are important for the accuracy of XA spectra in liquid solution.^29^

Asymmetry in the solvation environment around a symmetric molecule influences many degrees of freedom of the solute and can be important for chemical recognition and reactivity. The fluctuations in the asymmetry around the aqueous nitrate ion have been probed in potassium nitrate with infrared and ultraviolet–visible (UV–vis) spectroscopy.^30^ The asymmetry in hydrogen bonding in liquid water has been investigated in XA and X-ray emission spectroscopy.^9,10,12^ Asymmetry in hydrogen bonding has also been observed in aqueous p-Benzoquinone by UV–vis spectroscopy.^31^ In all these studies, theoretical analysis using quantum chemistry calculations and molecular dynamics or Monte Carlo simulations have been essential ingredients. We use a conceptually similar approach in our current study of symmetry breaking hydrogen bond fluctuations in the hydration shell around ammonia.

The nitrogen K-edge XA spectrum of 1 M aqueous ammonia is presented in Figure 1. It is of unprecedented quality and shows a clear splitting of the main-edge into a sharp 402.75 eV feature and somewhat broader feature at 403.25 eV, above the pre-edge at 401.2 eV. We notice that this splitting was not unambiguously detected in previous measurements in transmission mode^13^ and not seen in measurements with partial fluorescence yield.^17^ In relation to previous measurements in transmission mode,^13^ our spectra have higher signal-to-noise ratio. Despite a slightly lower energy resolution than that reported in ref (13)., we can clearly observe a main-edge splitting. The pre-edge and main-edge are well separated from the broad postedge feature at 408 eV and according to previous calculations they reside below and near the conduction band minimum of the solution,^13^ respectively. Hence, we assign them to excitations of N 1s (1a_1_) core–electrons into the 4a_1_ and 2e orbitals of NH_3_ with C_3v_ symmetry, respectively. We propose that the splitting of the main-edge is direct evidence of symmetry breaking arising from solute–solvent interactions that induces a lifting of degeneracy of the 1a_1_ → 2e transitions. Symmetry breaking influences core-excitations, and previously the appearance of weak pre-edge transitions in the nitrogen K-edge XA spectrum for the aqueous ammonium ion (with intrinsic *T*_d_ symmetry) has been ascribed to symmetry breaking solute–solvent interactions.^13^ However, in ammonia there is possibly a stronger sensitivity since the intense 1a_1_ → 2e transitions are affected. In future experiments, we could attempt to quantify the degree of asymmetry as a function of temperature and concentration, but in this paper we focus on a theoretical analysis of the spectral sensitivity.

**Figure 1:**
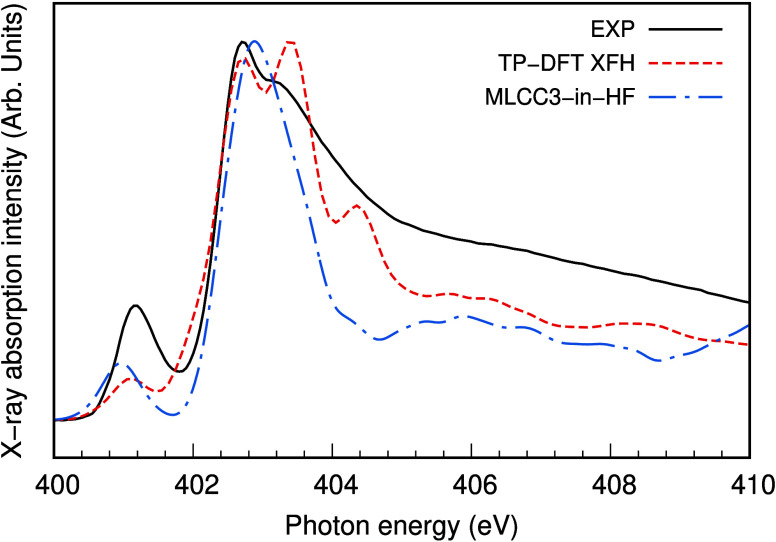
Comparison of experimental and simulated nitrogen K-edge XA spectra of NH_3_(aq). The experimental NH_3_(aq) 1 M XA spectrum in the vicinity of the N 1s ionization threshold was recorded at *T* = 25.2 °C in the setup described in ref (32). The position of the resonances: The pre-edge at 401.2 eV energy was calibrated against Ekimova et al. *J. Am. Chem. Soc.* 139 (2017) 12773.^13^ The main-edge is split into two features at 402.75 eV and at 403.25 eV, respectively, and the postedge is a very broad feature centered around 408 eV. The simulated TP-DFT XFH spectrum is reproduced from Ekimova et al., *J. Am. Chem. Soc.* 139 (2017) 12773,^13^ and the simulated MLCC3-in-HF spectrum is a reconvolution of the data presented in Folkestad et al., *J. Chem. Theory Comput.* 20 (2024) 4161.^27^

Nitrogen K-edge XA spectra of aqueous ammonia and ammonium have previously been analyzed with quantum chemistry at the TP-DFT XFH^13^ and MLCC3-in-HF^27^ levels of theory. In Figure 1, the simulated XA spectra of aqueous ammonia are reproduced in comparison to the new measurements. The calculated splitting of the 1a_1_ → 2e transition is seen as an asymmetry in the MLCC3-in-HF spectrum^27^ whereas two distinct components are obtained with the TP-DFT method.^13^ We notice that there are indications of a splitting in the MLCC3-in-HF spectrum but only when the convolution scheme is less broad (see Figure S8 in ref (27)) than for comparison to experimental data. This difference might be explained by the fact that the multilayer coupled cluster model prohibits certain types of orbital mixing. However, the TP-DFT and CC3 methods are very different computational schemes and we will focus on similarities in the results. In the TP-DFT XFH spectrum, the uniform convolution scheme, described in the Computational methods Section, also produces other features seen at 404.5 eV, 406 and 408.5 eV which is likely artificial and due to limitations in configurational sampling and local atomic orbital basis sets. The possible realism in these features will be explored in future investigations with more accurate models and different basis sets. The precise choice of convolution does not have physical rationale, but it is likely smaller than the combined effect of lifetime broadening, vibrational broadening, and experimental broadening. We notice that there is a weak dependence of the resolution of the splitting on the convolution scheme, which has been adapted to approximately match to the experiment for the single 1a_1_ → 4a_1_ transition in the pre-edge peak.

We focus the analysis below on the main-edge splitting in the TP-DFT XFH spectrum with features at 402.6 eV and at 403.4 eV, respectively. The intensity of the high-energy feature is exaggerated in comparison to experiment, and the magnitude of the splitting of 0.5 eV is overestimated in simulations (0.8 eV). In both respects, the disagreement is a combined effect of shortcomings of the TP-DFT XFH method and of the AIMD model. In the AIMD simulations, we employed an approximate DFT exchange-correlation functional and nuclear quantum effects were neglected.

The theoretical XA spectra in Figure 1 result from sampling many different configurations with varying hydrogen bond distances and other fluctuations in coordination of ammonia and in intermolecular geometries. To systematically monitor the effect of asymmetry in the hydration shell around ammonia, a microsolvated model cluster of NH_3_(H_2_O)_3_ was designed in *C*_*3*_ symmetry as depicted in Figure 2. The nitrogen K-edge XA spectra for both the TP-DFT XFH and CC3 methods of the fully symmetric cluster is shown with a solid black curve in Figure 2. The cluster is an idealized model of hydration, being symmetric and lacking a hydrogen bond donating water molecule, which has been shown to be present in aqueous solution.^17^ Because of the idealized geometry, the pre-edge region looks different from the solution data, but we focus on the main-edge region. Corresponding spectra for inward and outward displacement of one of the NH···O hydrogen bond distances are presented in dashed red and blue curves, respectively. They show a redistribution of intensity in the split feature in the main-edge region and a gradually increasing splitting in both directions. There are several spectral features, but given thermal fluctuations yielding elongated weak donating hydrogen bonds, we can understand why the main-edge splitting is visible for TP-DFT XFH and not for CC3.

**Figure 2:**
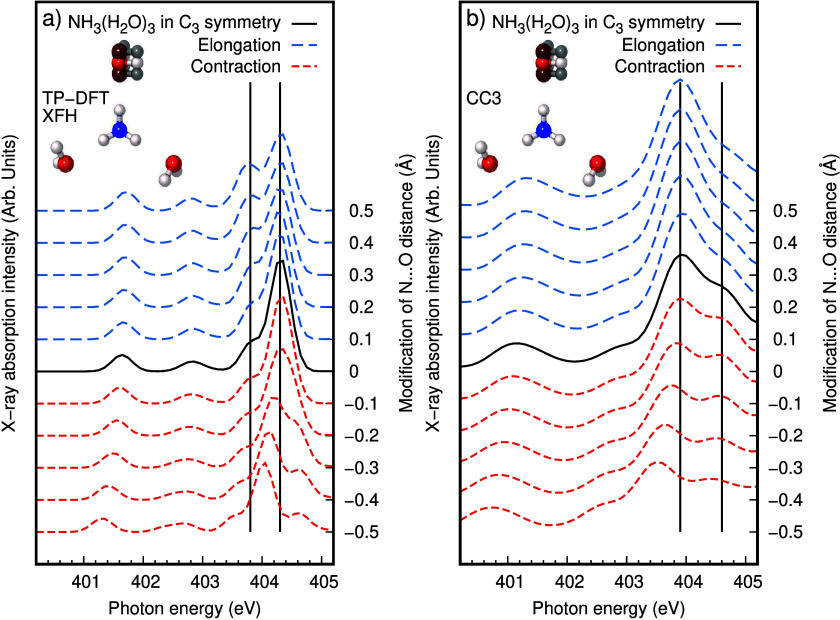
Spectral response in nitrogen K-edge X-ray absorption spectra to symmetry breaking in an idealized NH_3_(H_2_O)_3_ cluster. The inserted ball-and-stick model shows a rigid scan breaking the *C*_3_ symmetry by varying one NH···O hydrogen bond distance. Spectra with intensity associated with the left *y*-axis are simulated for the rigid scan with (a) the TP-DFT XFH method and (b) the CC3 method. When the symmetry is broken as denoted on the right *y*-axis, the X-ray absorption main-edge at 403–405 eV is gradually split. In both methods, the idealized cluster model has an additional spectral feature around 402.5 eV, not seen in the fully solvated model.

In the soft X-ray regime of nitrogen K-edge XA spectroscopy, the dipole approximation can be employed and for each discrete transition from the calculations, the transition dipole moment of the core-excitation can be used to decompose the intensity into Cartesian components (X,Y,Z). In Figure 3, we decompose the XA spectra from the rigid scan in the idealized cluster from Figure 2 into Cartesian components. This is a useful technique to learn more about the symmetries of orbital contributions to the spectrum also in solution.^17,33^ Disregarding the pre-edge region and focusing on the main-edge region around 404 eV, we can, in the absence of other degrees of freedom present in the solution, better purify splitting in the Y and X components perpendicular to the molecular *C*_*3*_ symmetry axis (along Z) and we can clearly identify the contributions from the short and long hydrogen bonds. Because of *C*_*3*_ symmetry in the idealized NH_3_(H_2_O)_3_ cluster, the Y and X components are equal in the absence of distortion. Even though the TP-DFT XFH and CC3 methods show differences in splitting and intensities, they exhibit similarities in the structural dependence in the spectrum. The Y main-edge component, which is associated with the varying hydrogen bond distance, shifts in energy by −0.9 eV/Å accompanied by an increase of intensity, and in addition loss of intensity in the feature around 406 eV, for increasing distance. Anticipating the analysis of the solution spectra below, we also notice that we reversely could use the direction and magnitude of the transition moments to analyze distortions in the complex.

**Figure 3:**
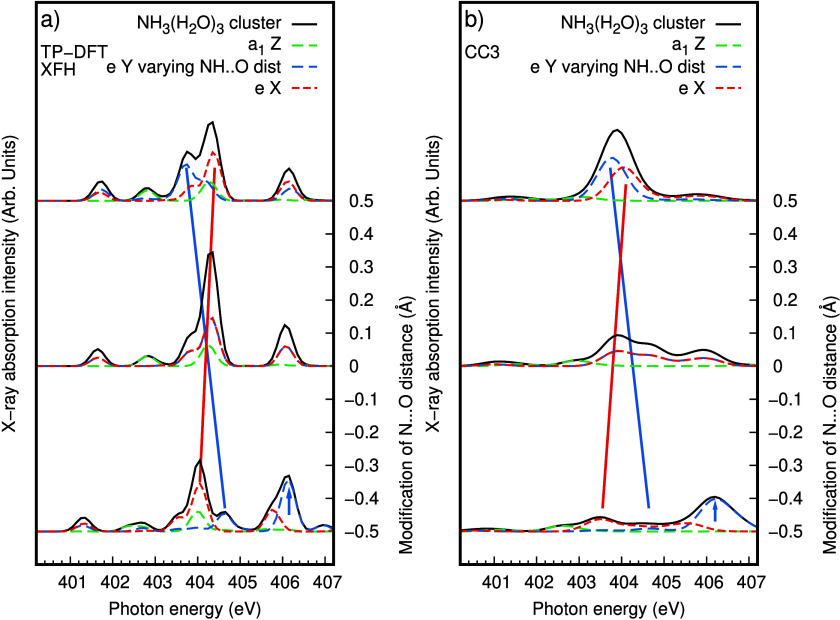
Spectral decomposition of the simulated nitrogen K-edge XA (a) TP-DFT XFH and (b) CC3 spectra for the distorted NH_3_(H_2_O)_3_ cluster. The spectral components of the spectra are determined in a molecular frame of the NH_3_ solute. In the cluster, the molecular *C*_3_ axis Z is defined as the sum of the N–H vectors, and the X direction as cross product between the Z and the N–H vector with the varying hydrogen bond (from NH_3_ to the water solvent). Hence, the Y component of the XA spectrum can be associated with the varying donating hydrogen bond. The transitions in the Z component are associated with a_1_ orbital symmetry, and in the Y, X components with e symmetry.

Having established that the observed splitting of the main-edge can be reproduced in the TP-DFT XFH method and that it can be related to asymmetry in hydrogen bonding, we proceeded to investigate the correlation between electronic and nuclear degrees of freedom in the simulated XA spectrum of aqueous ammonia. When sampling over many different configurations from the AIMD simulations, with the NH_3_ molecule in different orientations, we can gain more insight by averaging the decomposed XA spectral components in the molecular frame of the probed molecule. Because of the C_3v_ symmetry of ammonia, we can naturally define the molecular (C_3_) symmetry axis to be along Z in the molecular frame, but still have to make a choice concerning the orientation of the Y, X directions. We employ this freedom to investigate three different molecular frames defined either by nuclear degrees of freedoms in the solvent–solute interactions or by the electronic core-level transitions. The molecular frames are described in detail in the Computational methods Section. In molecular frames I and II, the Y component can be associated with shortest and longest donating hydrogen bond from ammonia, respectively. In molecular frame III inspired by insights from Figure 3, the Y component is associated with the direction of the strongest transition moment in the lower main-edge peak.

In a zoom-in on the main-edge region in Figure 4, the decomposition of the total XA (TP-DFT XFH) spectrum into Cartesian components is displayed for molecular frames I–III. The pre-edge peak, being of 1a_1_ → 4a_1_ character, is in all three cases captured by the X component, which further up in energy is comparably weak. In none of the frames, we could completely purify the split main-edge peaks into separate components. However, as expected from many previous studies of hydrogen bonds probed by XA spectroscopy,^9,34^ the contribution from short hydrogen bonds is blue-shifted relative to that of longer hydrogen bonds. Hence, the Y component has a larger contribution at 403.4 eV than at 402.6 eV in molecular frame I corresponding to the shortest hydrogen bond, and the other-way around in molecular frame II corresponding to the longest hydrogen bond. (Decomposition of the MLCC3-in-HF spectrum, presented in Figure S2 in the Supporting Information, molecular frames I–III exhibits the same trends as the TP-DFT XFH spectrum.)

**Figure 4:**
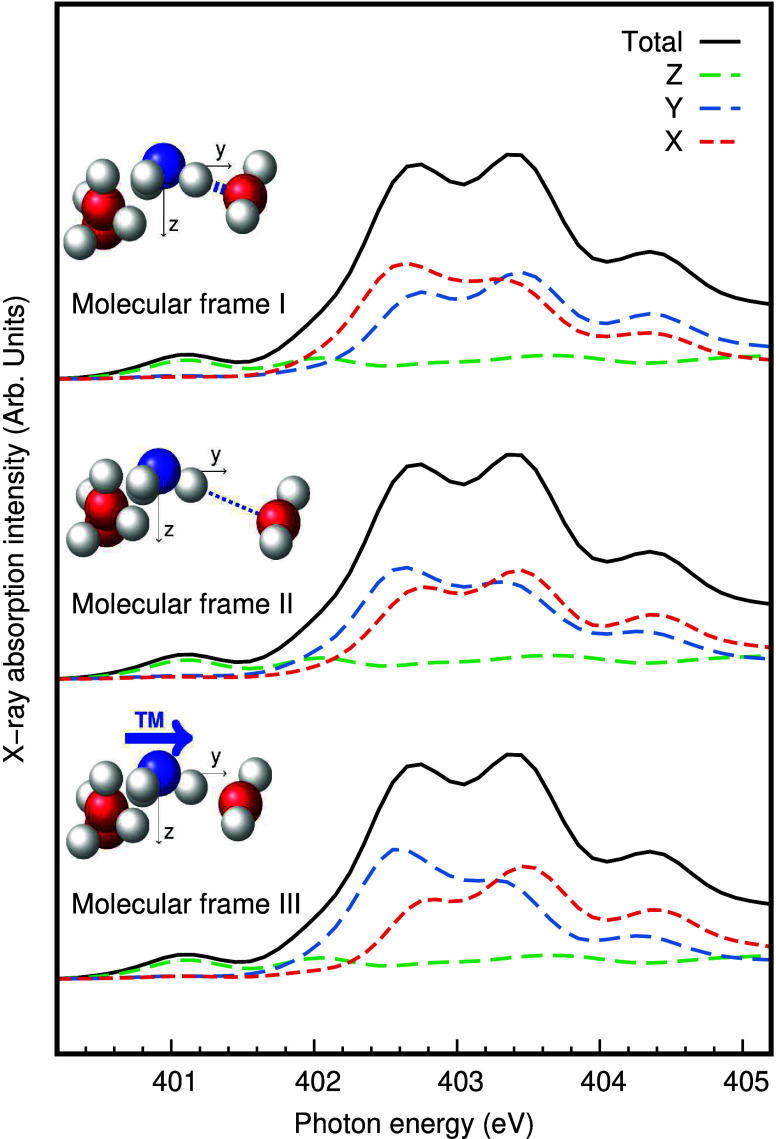
Decomposition of the simulated nitrogen K-edge X-ray absorption TP-DFT XFH spectrum of NH_3_(aq). The decomposition is based on a reanalysis of the previously published data.^13^ The spectra are averaged in different definitions of the molecular frame of the NH_3_ solute. In all three definitions, the molecular *C*_3*v*_ axis Z is defined as the sum of the N–H vectors, but different conventions of the Y direction are employed to evaluate the sensitivity to the symmetry breaking. Molecular frame I and II are based on geometric criteria and the spectral Y component is associated with the N–H vector involved in shortest (I) and longest (II) hydrogen bond. (The distributions of short and longest N···O_w_ distances in the hydration shell of ammonia are presented in Figure S1b.) Molecular frame III is instead derived from electronic considerations, in which the Y component is associated with the strongest transition moment in the region of the lower main-edge peak.

We notice that the geometric definitions in molecular frames I–II are equally (un)successful in purifying the contributions to the split features. The spectroscopic definition employed in molecular frame III is more suitable, but less fruitful since it does not give structural insight. Importantly, however, we see that also for molecular frame III, there is a contribution of component Y to both the 402.6 and 403.4 eV features, which should not be possible if each configuration would results in only two features of e symmetry with perpendicular transition moments. Hence, it indicates that there is a distribution of different local environments with different asymmetry contributing to the total signal.

To conclude the analysis in Figure 4, we have explored angular decomposition of the simulated nitrogen K-edge XA spectrum of aqueous ammonia. We can deduce that the splitting of the main-edge is due to perpendicular transitions of e symmetry and we establish a relation to hydrogen bonding distances. However, to reach further insight, we need to analyze the degree of asymmetry around the ammonia solute. The radial distribution function g(r) of N···O_w_ from the AIMD simulation is shown in Figure S1, with the individual contributions of the closest O_w_ accepting a hydrogen bond for each of the N–H bonds are highlighted in Figure S1b, such that N–H_i_, *i* = 1, 2, 3 are ordered in increase hydrogen bond length. This shows the average instantaneous asymmetry around the solute which is responsible for the splitting in the main-edge of the nitrogen K-edge XA spectrum in Figure 1. As a measure of the asymmetry, the distributions of the ordered H···O_w_ distances are shown in Figure 5a together with the instantaneous difference between the shortest and longest distances in Figure 5b. There is a pronounced asymmetry in hydrogen bonding with a maximum in the distribution around 0.5 Å distortion. In Figure 5b, the vertical red lines separate regions for three classes of asymmetry to be used in the spectral analysis. The selected regions are arbitrarily defined, but have similar statistical weight.

**Figure 5:**
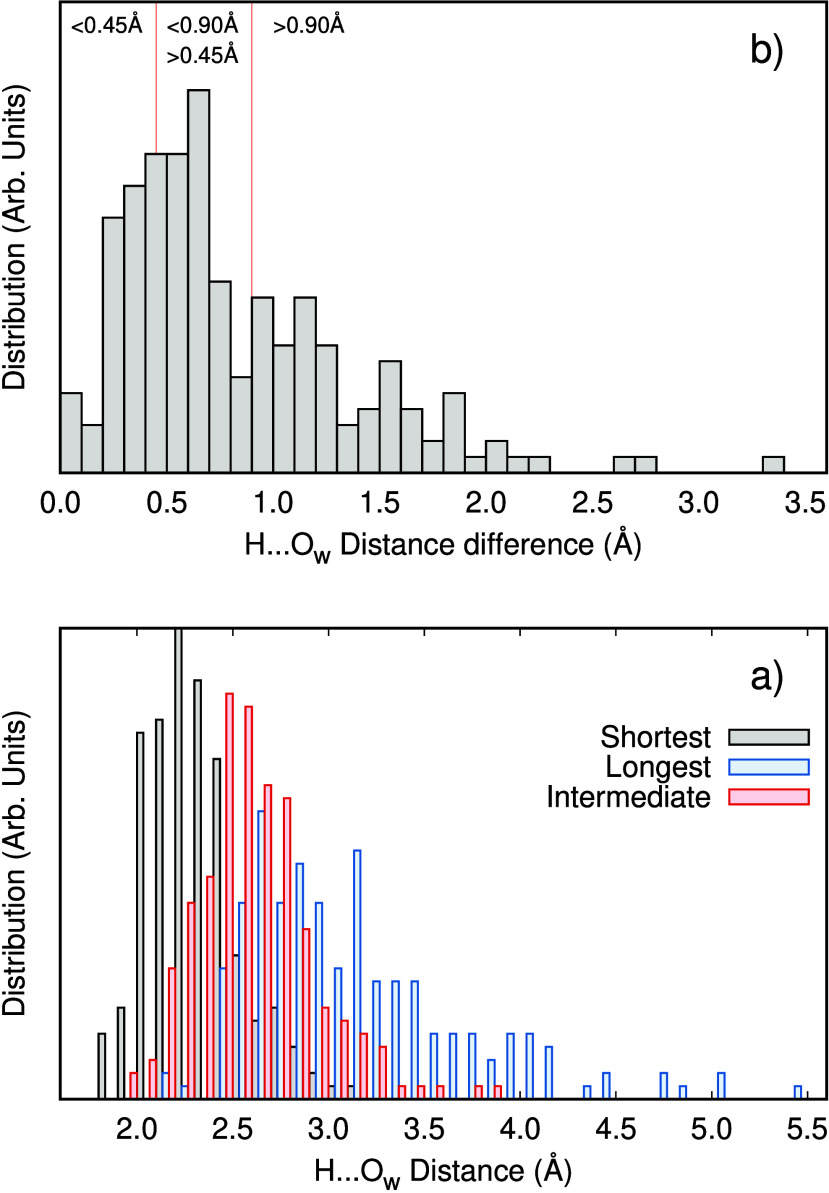
Distribution of the three (N-)H···O_w_ distances in the AIMD simulation of aqueous ammonia. (a) Distributions of distances in increasing order. (b) Distributions of difference Δ(H···O_w_) between longest and shortest of the three (N-)H···O_w_ distances.

Finally, we relate back to the spectral decomposition in Figure 4 and make an attempt to quantify the spectral response to the hydrogen bond asymmetry shown in Figure 5. The nitrogen K-edge XA (TP-DFT XFH) spectrum of aqueous ammonia in molecular frame I is subdivided into different classes of asymmetry according to the regions in Figure 5b. In the angular analysis in Figure 6, we see how the Y and X components depend on the asymmetry, being almost equivalent for small asymmetries. With increasing asymmetry, the contribution to the high (low) energy feature gets increasingly pure for the Y (X) component. The Z component is relatively insensitive to the degree of asymmetry. However, even at large Δ(H···O_w_) asymmetry in the hydration shell, the resulting sum of the components only show a small increase in the splitting of the total spectrum. Neither the Y component nor the X component purely contribute to one of the peaks in the main-edge. This could indicate that we have not found an optimal definition of the molecular frame. Combined with the insights from the decomposition in molecular frame III, we conclude that there is mixing between the solute and solvent molecular orbitals, which contributes to the splitting of the molecular e transitions. However, scanning through the individual spectra underlying the sampling, we notice that averaging hides some of the complexity of the spectral response to structural fluctuations. Contributing to the average spectrum, there are individual configurations with spectra of very different spectral shapes and shifts in the peak positions. E.g. spectra that lacks a significant contribution to the lower peak in the split main-edge. This is at least part of the explanation of our failure to find a molecular frame that can perfectly separate the split main-edge into different angular components.

**Figure 6:**
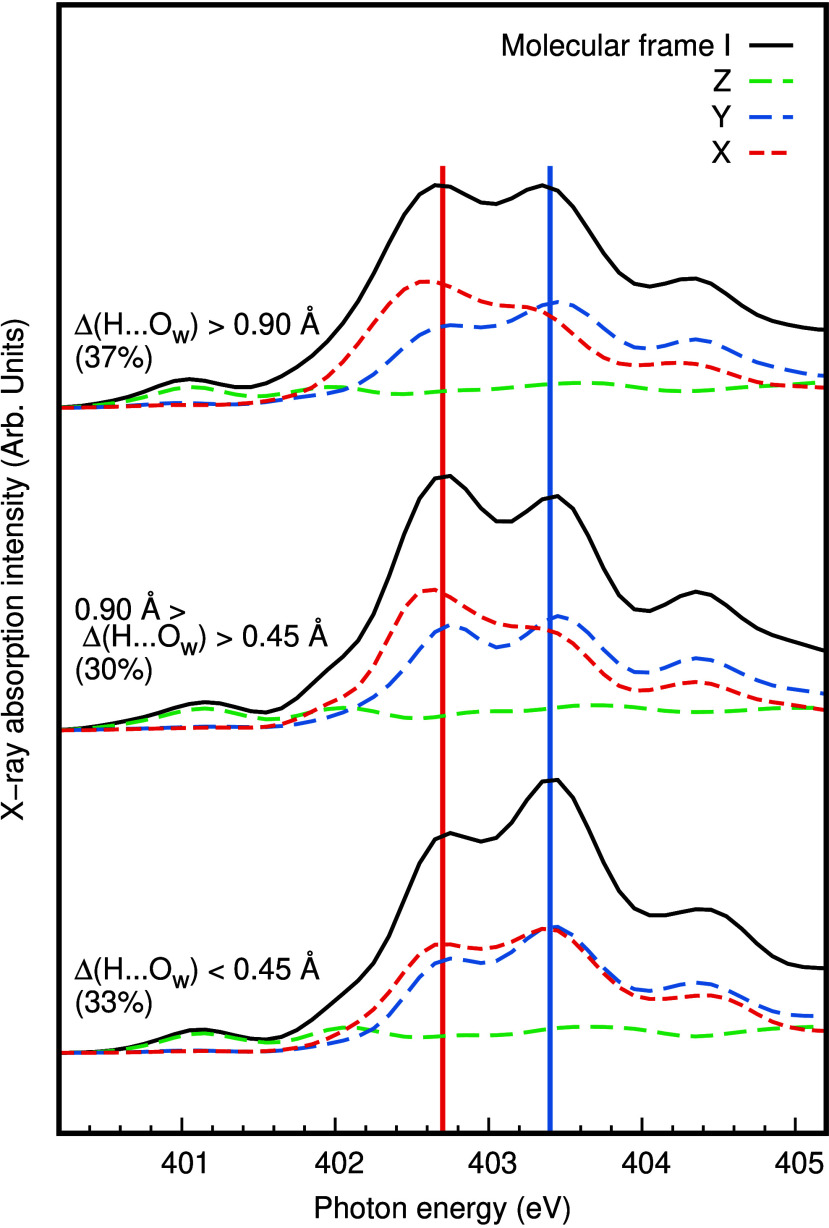
Further asymmetry-related decomposition of the simulated nitrogen K-edge XA TP-DFT XFH spectra of aqueous ammonia. Different classes of asymmetry, defined according to Figure 5b, allow us to corroborate the trend seen in the idealized NH_3_(H_2_O)_3_ cluster in Figure 3. Spectra are presented in molecular frame I as defined in Figure 4.

In new accurate measurements, we have discovered a splitting in the main-edge of the nitrogen K-edge X-ray absorption spectrum of aqueous ammonia. This experimental observation is reproduced and further analyzed in spectrum simulations. XA spectroscopy is an element specific probe of the local electronic structure which in turn depends on the molecular structure. In aqueous solution, the XA signal is sensitive to hydrogen bonding interactions,^9,10^ and for ammonia, we have investigated at different levels of theory how the spectrum is influenced by interactions with surrounding solvent.^17,26,27^

In this study, we show how the splitting in the XA main-edge for aqueous ammonia can be related to asymmetry in donating hydrogen bonds around the ammonia solute. There is a complex dependence of the XA spectrum on the local coordination and also more long-range interactions,^26^ but angular analysis of the simulated XA spectra in the molecular frame of ammonia has allowed us to assign the spectral features in the main-edge and to explore how the asymmetry in hydrogen bonding influences the splitting. The residual splitting, seen also for small asymmetry, indicates that many different spectral shapes and orbital mixing between the solute with the surrounding solvent environment partially contribute to the splitting. Investigations of XA spectra of of amine groups^18,35^ could be used to probe asymmetries in hydrogen bonding relevant for understanding the solvation of functional groups in biomolecules. We are also looking into the temperature dependence in the XA spectra of ammonia which could give further insight in the degree of asymmetry related to fluctuations in the hydrogen bond network. We have explored two intrinsically different protocols for XA spectrum simulations, TP-DFT XFH and MLCC3-in-HF, sampled over the same set of configurations from an AIMD simulation. Although, we notice quantitative differences between the simulation protocols, we see qualitative similarities in the angular analysis supporting the proposed mechanism for the observed splitting. The splitting of the main-edge at the TP-DFT XFH level is overestimated (0.8 eV) in comparison to experiment (0.5 eV), whereas there are not sufficiently distinct peaks at the MLCC3-in-HF level to show any splitting. From previous quantum mechanics molecular mechanics (QM/MM) calculations varying the cluster size of the QM cluster at the expense of the surrounding MM region, we notice variations in the main-edge splitting and changes in the postedge feature in the QM/MM models pointing to finite size effects with respect to the QM region^26^ (see red curves in Figure S11 in ref (26).). In addition, since the average XA spectrum sampled from the AIMD can be sensitive to the limitations of the approximations in DFT, we also sampled an AIMD simulation performed with the more recently developed “Strongly Constrained and Appropriately Normed” (SCAN) DFT functional.^36^ Due to the computational cost, this check was only performed on the TP-DFT XFH level and not on the MLCC3-in-HF level. The details about the SCAN AIMD simulation are given in the SI together with the resulting XA spectrum in Figure S3, which shows an angular decomposition closely resembling that in Figure 4 and a weak dependence on the DFT approximation.

## Experimental and Computational Methods

The nitrogen K-edge XA spectrum of 1 M aqueous ammonia was measured with photon energy steps of 0.2 eV for the range 398–399.5 eV, 0.05 eV for the range 399.5–404 and 0.1 eV for the range 404–417 eV. The measurements were performed at the BL3U beamline, UVSOR-III Synchrotron, Institute for Molecular Science (IMS), Japan, covering the 60–800 eV photon energy range with a resolving power setting of E/ΔE = 2000 at hν = 400 eV. The spectra were recorded in transmission mode using a special cell whose liquid thickness between two SiC membranes (of 100 nm thickness each) was optimized by controlling the flowing helium pressure inside a dedicated vacuum chamber. The temperature of the liquid in the cell was controlled using an Eyela-chiller NCC-3000, leading to a measured value of 25.2 °C. Photon energy calibration was performed by recording the nitrogen K-edge XA spectrum of a ProLINE polymer film. A detailed description of this setup is given in ref (32). An ammonia aqueous solution of 25% in mass was purchased from Sigma-Aldrich and diluted with deionized water to reach a concentration of 1M.

For assignment of the observed main-edge splitting in the new experimental data presented in this study, we performed an angular analysis of the simulated nitrogen K-edge XA spectrum of aqueous ammonia. The theoretical investigation involved further analysis of previously published spectrum simulations^13,27^ and also entailed modeling the spectral response to systematic distortions from *C*_*3*_ symmetry of an idealized NH_3_(H_2_O)_3_ cluster. Derivation of Cartesian components of transition dipole moments of the core excitations in the molecular coordinate system of the NH_3_ solute allows for investigation of electronic asymmetry resulting from fluctuations in the hydrogen bond network.

The configurational sampling of XA spectra of aqueous ammonia was performed on 195 configurations from an AIMD simulation with the mixed Gaussian and plane wave (GPW) method in the CP2K program.^37,38^ The dispersion-corrected BLYP-D3 functional^13^ and DZVP basis sets for Goedecker-Teter-Hutter pseudopotentials^39,40^ were employed. Further details about the AIMD simulation are given in the original publication.^13^ To investigate the spectral response to structural distortions, both the AIMD simulation trajectory and an idealized cluster model were analyzed. A microsolvated NH_3_(H_2_O)_3_ cluster was geometry optimized with the BLYP-D3 functional in Gaussian 09.^41^ The cluster geometry is constrained to *C*_*3*_ symmetry and an N–H···O_w_ angle of 179.95° using a Z-matrix and it is not a local energy minimum but serves the purpose for investigating symmetry breaking. A rigid scan of symmetry breaking distortions around the optimized geometry with N···O_w_ of 3.329 Å was performed by varying one N···O_w_ distance [-0.5 Å:+0.5 Å] as depicted in Figure 2.

At the DFT level of approximation, nitrogen K-edge XA spectrum simulations were obtained with the BLYP functional in the TP-DFT XFH approach^25^ as implemented in the Gaussian and augmented plane wave (GAPW) method^42^ in CP2K.^38^ For nitrogen, oxygen, and hydrogen atoms, we used the ANO-L basis set, 6–311Gxx, and 6–311++G2d2p basis sets, respectively.^17^ Further details about the spectrum simulations are given in the original publication.^13^ The resulting nitrogen K-edge XA spectrum of aqueous ammonia is reproduced in Figure 1. The TP-DFT XFH approach was also used for the XA spectrum simulations of a rigid scan in the isolated NH_3_(H_2_O)_3_ cluster in a cubic simulation cell with edge 20 Å using a Poisson solver for isolated systems.^43^ For comparison to the experimental data, all TP-DFT XFH spectra were shifted by −10.95 eV and convoluted with a Gaussian with a full width at half-maximum of 0.4 eV.

XA spectra of aqueous ammonia are reproduced from a previous study using MLCC3-in-HF^27^ and additionally corresponding CC3 calculations were performed on the isolated NH_3_(H_2_O)_3_ cluster. In the MLCC3-in-HF approach, each configurations sampled from the simulation is partitioned into an active region, described with MLCC3,^44−46^ and an environment described by a frozen Hartree–Fock density. This partitioning of the molecular system is obtained by calculating localized Hartree–Fock orbitals, which can be assigned to the active region or the environment. Only the active orbital space is included in the coupled cluster calculation. The MLCC3 approach requires a further partitioning of the active orbital space. In MLCC3, the triple excitations in the cluster operator are restricted to a subset of the orbitals, and the excitation amplitudes are determined perturbatively.^47^ A nitrogen K-edge XA spectrum is calculated with MLCC3-in-HF by considering ammonia and the four closest neighboring water molecules at the MLCC3 level of theory. An aug-cc-pVTZ basis set is used for ammonia, aug-cc-pVDZ for the four closest water molecules, and cc-pVDZ is used for the water molecules described by a frozen Hartree–Fock density. To partition the system, we use Cholesky occupied orbitals^48^ and projected atomic (virtual) orbitals.^49,50^ The triple excitations in the cluster operator are restricted to an active space obtained from the correlated natural transition orbitals^51^ obtained with CCSD-in-HF. See refs (27) and (29) for a detailed description of the approach and of the cluster models cut out from the simulations. Due to the high accuracy, no energy shift is required for the CC3 or MLCC3-in-HF calculations.

In order to analyze the simulated nitrogen K-edge XA spectrum of aqueous ammonia, which reproduces the splitting of the main-edge observed in the experimental measurements, we performed an angular analysis by decomposing the transition dipole moments into their Cartesian components. Because of isotropic reorientation of the ammonia molecule in solution, it is only meaningful to perform this analysis in a molecular frame of the solute. The molecular *C*_*3*_ symmetry axis of ammonia is a natural reference direction (Z) also in solution, which for each configuration along the AIMD can be obtained as the normalized sum of the three N–H_N_ vectors. However, in defining a molecular coordinate system we have a freedom in defining the perpendicular (Y and X) directions, and hence we hence considered different molecular frames which have in common that Z is along the *C*_*3*_ symmetry axis.

In molecular frame I, the X axis is defined as the cross product between the Z axis and the N–H vector with the shortest hydrogen bond, consequently the Y component of the XA spectrum can be associated with the shortest donating hydrogen bond from NH_3_ to the water solvent. The distance measure is determined from (N-)H···O_w_ distances with angles N–H···O_w_ > 130°. Molecular frame II is analogously defined in terms of the N–H vector with the longest hydrogen bond which is associated with the Y component. Hence, both molecular frames I and II are defined in terms for structural degrees of freedom, but we also wanted to explore a definition based on the electronic structure. To capture the character of the lower main-edge peak in the TP-DFT XFH spectrum in Figure 1, molecular frame III is instead defined in terms of the strongest core-excitation in the energy region below 403 eV. The X axis is defined as the cross product between the Z axis and a corresponding transition moment, and hence the direction of the strongest transition moment can be associated with the Y component. Pictorial representations of the molecular frames are shown as insets in Figure 4.

## Data Availability

The data sets generated and analyzed during the current study can be accessed on Zenodo10.5281/zenodo.14513943 (geometries, example CP2K inputs, and TP-DFT XFH and CC3 spectra). Further information is available from the corresponding authors on reasonable request.

## Supporting Information Available

Complementary information on radial distribution functions around the ammonia molecules and decomposition of the MLCC3-in-HF simulated nitrogen K-edge XA spectrum of NH_3_(aq) and of the TP-DFT XFH spectrum sampled from the AIMD simulation with the SCAN functional (PDF)
Transparent Peer Review report available (PDF)
