# Supplementary material for: Symmetry Breaking around Aqueous Ammonia Revealed in Nitrogen K-edge X-ray Absorption
Source: J Phys Chem Lett. 2025 Mar 27;16(14):3411–9. doi: 10.1021/acs.jpclett.4c03625 (PMC11998083; doi:10.1021/acs.jpclett.4c03625)
Supplement: Supplementary file 1 — jz4c03625_si_001.pdf [file jz4c03625_si_001.pdf]

Symmetry Breaking around Aqueous Ammonia Revealed in Nitrogen K-edge X-ray Absorption

Michael Odelius,*,† Sarai Dery Folkestad,‡ Thanit Saisopa,¶ Yuttakarn Rattanachai,¶ Wutthigrai Sailuam,§ Hayato Yuzawa,|| Nobuhiro Kosugi,|| Alexander C. Paul,‡ Henrik Koch,‡ and Denis Céolin*,⊥

†*Department of Physics, Stockholm University, 10691 Stockholm, Sweden*

‡*Department of Chemistry, Norwegian University of Science and Technology, NTNU, 7491 Trondheim, Norway*

¶*Department of Applied Physics, Faculty of Sciences and Liberal Arts, Rajamangala University of Technology Isan, Nakhon Ratchasima 30000, Thailand*

§*Department of Applied Physics, Faculty of Engineering, Rajamangala University of Technology ISAN (Khon Kaen Campus), Khon Kaen 40000, Thailand*

||*UVSOR Synchrotron Facility, Institute for Molecular Science, Okazaki 444-8585, Japan*

⊥*Synchrotron SOLEIL, L'Orme des Merisiers, BP 48, St Aubin, 91192 Gif sur Yvette, France*

E-mail: odelius@fysik.su.se; denis.ceolin@synchrotron-soleil.fr

Supplementary information

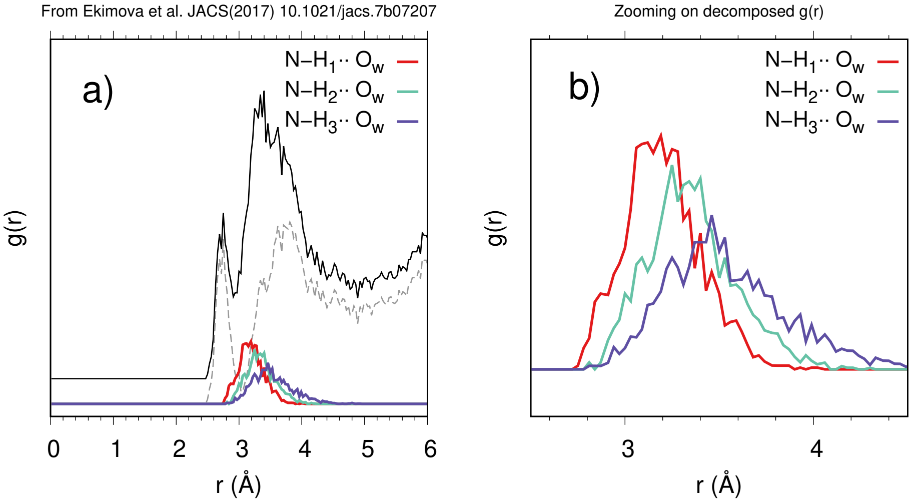

Figure S1: Radial distribution function g(r) of N$\cdots$O$_w$ hydrogen bonding in aqueous ammonia. a) Data reproduced from Ekimova *et al.* J. Am. Chem. Soc. 139 (2017) 12773.[1] The solid black line is the total N$\cdots$O$_w$ g(r) and the gray line corresponds to the hydrogen bond from water to ammonia. b) Zoom in on the partial ordered N-H$\cdots$O$_w$ radial distribution functions showing the asymmetry around the ammonia solute.

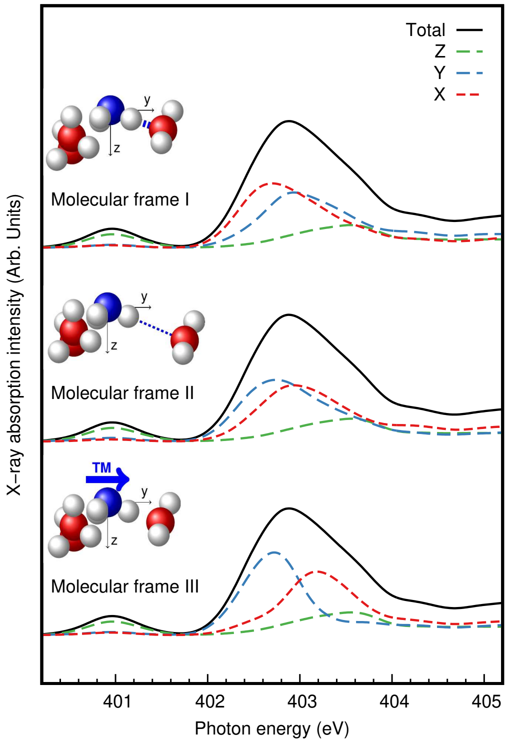

Figure S2: Decomposition of the MLCC3-in-HF simulated nitrogen K-edge XA spectrum of NH_3(aq) from Figure 1 employing molecular frames I-III as defined in Figure 4.

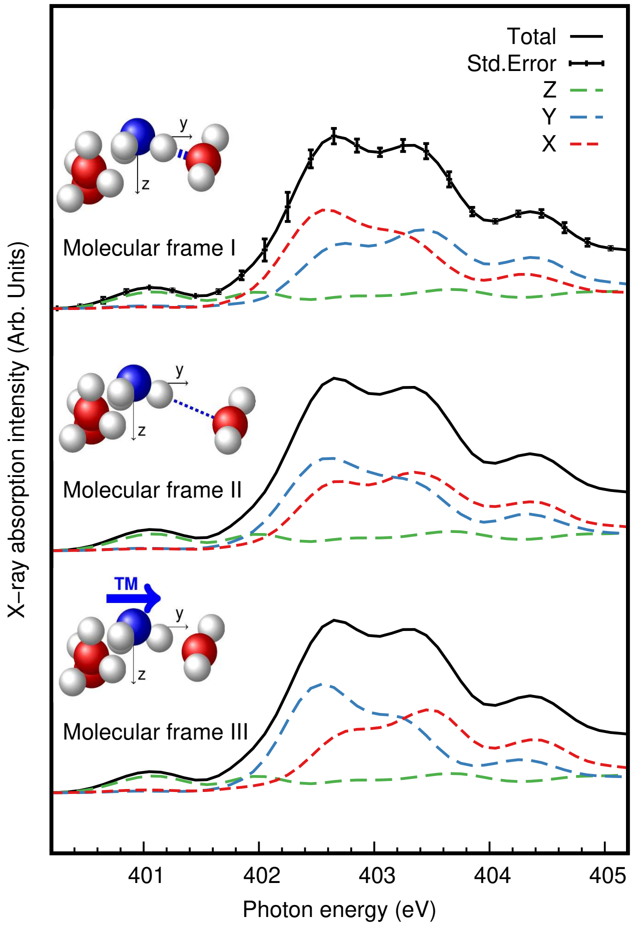

Figure S3: Decomposition of the simulated nitrogen K-edge X-ray absorption TP-DFT XFH spectrum of NH_3(aq) from the AIMD simulation with the SCAN functional. The details of the decomposition are given in the analogous presentation for the BLYP-D3 AIMD simulation in Figure 4. The graph also includes the standard error for the sampling, obtained by block-averaging into 5 blocks.

To evaluate whether the XA spectrum sampled from the trajectory of the AIMD simulation is sensitive to the choice of exchange-correlation functional in the AIMD simulations, we performed an AIMD simulation with the strongly constrained and appropriately normed (SCAN) DFT functional.[2] The SCAN AIMD simulation was initialized at the end of the BLYP-D3 AIMD simulation, performed at the same conditions, equilibrated for 15 picoseconds, and then simulated for 30 picoseconds. A sampling of altogether 200 spectra was used for the XA spectrum presented in Figure S3, in which we repeat the angular analysis performed in Figure 4 but on spectra from the SCAN trajectory.

References

(1) Ekimova, M.; Quevedo, W.; Szyc, Ł.; Iannuzzi, M.; Wernet, P.; Odelius, M.; Nibbering, E. T. J. Aqueous Solvation of Ammonia and Ammonium: Probing Hydrogen Bond Motifs with FT-IR and Soft-X-Ray Spectroscopy. *J. Am. Chem. Soc.* **2017**, *139*, 12773–12783.

(2) Sun, J.; Ruzsinszky, A.; Perdew, J. P. Strongly Constrained and Appropriately Normed Semilocal Density Functional. *Phys. Rev. Lett.* **2015**, *115*, 036402.
