# Supplementary material for: Symmetry Breaking around Aqueous Ammonia Revealed in Nitrogen K-edge X-ray Absorption
Source: J Phys Chem Lett. 2025 Mar 27;16(14):3411–9. doi: 10.1021/acs.jpclett.4c03625 (PMC11998083; doi:10.1021/acs.jpclett.4c03625)
Supplement: Supplementary file 2 — jz4c03625_si_002.pdf [file jz4c03625_si_002.pdf]

jz-2024-03625m.R1

Name: Peer Review Information for "Symmetry Breaking around Aqueous Ammonia Revealed in Nitrogen K-edge X-ray Absorption"

First Round of Reviewer Comments

Reviewer: 1

Comments to the Author

The authors conduct a very detailed analysis of an XA spectrum of a 1M aqueous solution of NH3. They observe a substantial splitting in the main resonance which is attributed to hydrogen with the water solvent, which to me seem reasonable and consistent with existing literature that probes hydrogen bonding with XAS. The authors detailed computational analysis additionally allows an understanding of how the calculated spectral shape is influenced by terms of bond lengths, asymmetries and orbital interactions.

The series of computational approaches used appear robust (at least to a non expert). I appreciate the comparison of multiple computational methods in interpreting the data. I also appreciate the honesty of the authors regarding the challenges involved in conducting these sorts of advanced calculations. However, I am not a computational chemist and strongly believe that this article warrants a review from an expert on computational methods for simulating XAS.

From the scientific perspective, the authors certainly provide significant insight into this system. The assignment of hydrogen bonding features in x-ray spectroscopy to hydrogen bonding is something that requires a huge amount of care and this sort of detailed analysis is of value to the X-ray spectroscopy community. I therefore think the significance of this work is high enough to warrant publication in The Journal of Physical Chemistry Letters (pending reviews from experts in calculating X-ray spectra)

Reviewer: 2

Comments to the Author

The reviewed manuscript, entitled "Symmetry Breaking around Aqueous Ammonia Revealed in Nitrogen K-edge X-ray Absorption" by M. Odelius et al., reports on new, more precise/better resolved measurements of the X-ray absorption spectrum of aqueous ammonia recorded in transmission mode. The main novelty is the observation of splitting in the main edge of the spectra, which was not well resolved in previous studies, and its interpretation as a consequence of asymmetry in hydrogen bonding.

The experimental data indeed appear smoother than those reported in Reference 13. However, if both datasets were superimposed, the difference might not be substantial. In this context, could the authors provide an estimate of the experimental error bars?

The error estimate should be also provided to the XFH calculations. Since the data are extracted from a limited number of points, statistical error could play a role. In this context, it should be also clarified whether the Gaussian kernel with a FWHM of 0.4 eV was used purely as a smoothing procedure or if there is a physical rationale behind it, such as accounting for the 1s core-hole lifetime. In that case, Lorentzian might be more appropriate yet this is a minor issue.

Some of the data (or closely related results) were already presented in Reference 13. This study introduces new measurements and hybrid MLCC3-in-HF calculations. However, the latter calculations fail to reproduce the observed splitting. The authors attribute this discrepancy to the lack of a certain type of orbital mixing in the hybrid CC3 calculations. Yet maybe, the difference has a more fundamental cause, as very different qualitative features are observed even for the C3 symmetry cluster model—most notably, the flipped intensity of the two peaks. The CC3 data should probably be considered a benchmark for the cluster case.

If so, an alternative hypothesis should be considered: perhaps the CC3 calculations are actually correct, and the lack of agreement with the experiment arises from a biased sampling of aqueous ammonia. This possibility is plausible given the generally poor performance of DFT functionals for liquid water.

This raises the question of why the C3 symmetry cluster was chosen as a model, assuming the ab initio dynamics data are reliable. Reference 13 suggests that such configurations are not typical for ammonia in liquid water – or is this not the case? The manuscript contains references to the connection between the model system and the ab initio dynamics data (e.g., lines 13–18 on page 7), but these explanations appear somewhat cryptic.

On the other hand, adopting a simple model with high symmetry might offer the advantage of applying group theory to rationalize the observed splitting and the trends.

This study presents an interesting attempt to link molecular structure with X-ray absorption spectra of aqueous systems. It remains unclear whether this work fully capitalizes on the available data to establish a definitive relationship. While the connection between liquid structure and XAS seems clear, the precise nature of this relationship—and how best to model it—is not. I understand that additional data (e.g., measurements at different temperatures or using deuterated solutions) may not be available at this stage. Nevertheless, the interpretation should be presented with more caution.

Author's Response to Peer Review Comments:

Please see attached rebuttal letter

Notice that I included for both manuscript and for SI - versions with and without coloring the changes in the revision.

NH3aqXAS_JPCL_resubmit20250219.pdf

NH3aqXAS_JPCL_SI_resubmit20250219.pdf

NH3aqXAS_JPCL_resubmit20250219Color.pdf

NH3aqXAS_JPCL_SI_resubmit20250219Color.pdf

Revision: Manuscript ID jz-2024-03625m

To the editors of *The Journal of Physics Chemistry Letters*

Please find enclosed a revised version of the manuscript entitled *Symmetry Breaking around Aqueous Ammonia Revealed in Nitrogen K-edge X-ray Absorption* by Michael Odelius, Sarai Dery Folkestad, Thanit Saisopa, Yuttakarn Rattanachai, Wutthigrai Sailuam, Hayato Yuzawa, Nobuhiro Kosugi, Alexander C. Paul, Henrik Koch, and Denis C´eolin.

We appreciate the feedback from the reviewers and I have tried give appropriate response to the comments and also modified the manuscript accordingly. We would ask for the revision to be considered for publication as an article in The Journal of Physics Chemistry Letters.

We apologize that the resubmission was delayed, since we needed to perform additional AIMD simulations with a more modern exchange-correlation functional and associated sampling of X-ray absorption spectra.

On behalf of the authors, sincerely Yours

Michael Odelius
Department of Physics
Stockholm University, Stockholm, Sweden
Stockholm February 28, 2025

Editorial instructions

Format: Your revised manuscript must adhere to The Journal of Physical Chemistry Letters format.

Supporting Information: If the manuscript is accompanied by any supporting information for publication, a brief description of the supplementary material is required in the manuscript. The appropriate format is: Supporting Information. Brief statement in nonsentence format listing the contents of the material supplied as Supporting Information.

Funding Sources: Authors are required to report ALL funding sources and grant/award numbers relevant to this manuscript. Confirm all sources of funding for ALL authors relevant to this manuscript are included in BOTH the submission form and in the manuscript file to meet this requirement.

ORCID: Authors submitting manuscript revisions are required to provide their own validated ORCID iDs before completing the submission, if an ORCID iD is not already associated with their user profiles. This iD may be provided during original manuscript submission or when submitting the manuscript revision. You can provide only your own ORCID iD, a unique researcher identifier. If your ORCID iD is not already validated and associated with your user profile, you may do so by following the ORCID-related links in the Email/Name section of your user account. All authors are encouraged to register for and associate their own ORCID iDs with their user profiles. The ORCID iD will be displayed in the published article for any author on a manuscript who has a validated ORCID iD associated with their user account when the manuscript is accepted. Learn more at http://www.orcid.org.

Authors' response: We have revised the manuscript and supporting information according to the standards of The Journal of Physical Chemistry Letters, and we have gone through the author checklist.

Changes in revision: Section titles and sub-section titles were removed. Except for "Experimental and computational methods" after the conclusions

We also require a graphic for the "Table of Contents" with all submissions. We ask that you include a graphic immediately after the Abstract under the header "TOC Graphic." **Authors' response:** A TOC Graphic is included.

Changes in revision: Formatted according to the standards of The Journal of Physical Chemistry Letters.

Reviewers' comments and editorial instructions

Reviewer: 1

Recommendation: This paper represents a significant new contribution and should be published as is.

Comments: The authors conduct a very detailed analysis of an XA spectrum of a 1M aqueous solution of NH3. They observe a substantial splitting in the main resonance which is attributed to hydrogen with the water solvent, which to me seem reasonable and consistent with existing literature that probes hydrogen bonding with XAS. The authors detailed computational analysis additionally

allows an understanding of how the calculated spectral shape is influenced by terms of bond lengths, asymmetries and orbital interactions.

The series of computational approaches used appear robust (at least to a non expert). I appreciate the comparison of multiple computational methods in interpreting the data. I also appreciate the honesty of the authors regarding the challenges involved in conducting these sorts of advanced calculations. However, I am not a computational chemist and strongly believe that this article warrants a review from an expert on computational methods for simulating XAS.

From the scientific perspective, the authors certainly provide significant insight into this system. The assignment of hydrogen bonding features in x-ray spectroscopy to hydrogen bonding is something that requires a huge amount of care and this sort of detailed analysis is of value to the X-ray spectroscopy community. I therefore think the significance of this work is high enough to warrant publication in The Journal of Physical Chemistry Letters (pending reviews from experts in calculating X-ray spectra)

Authors' response: We appreciate the positive feedback on our study, and that it could be interesting for the readership of "The Journal of Physical Chemistry Letters". We have tried to further improve the presentation.

Reviewer: 2

Recommendation: This paper may be publishable, but major revision is needed; I would like to be invited to review any future revision.

Comments: The reviewed manuscript, entitled "Symmetry Breaking around Aqueous Ammonia Revealed in Nitrogen K-edge X-ray Absorption" by M. Odelius et al., reports on new, more precise/better resolved measurements of the X-ray absorption spectrum of aqueous ammonia recorded in transmission mode. The main novelty is the observation of splitting in the main edge of the spectra, which was not well resolved in previous studies, and its interpretation as a consequence of asymmetry in hydrogen bonding.

The experimental data indeed appear smoother than those reported in Reference 13. However, if both datasets were superimposed, the difference might not be substantial. In this context, could the authors provide an estimate of the experimental error bars?

Authors' response: In the first graph below, we superimposed the two recent datasets to highlight the presence of two structures in the main peak, which appear as an asymmetry in the spectrum of the reference 13 (black curve). The two XA spectra taken at the same temperature of 25.2°C are shown. They are identical and do not show intensity fluctuations (uncertainties) in the region of the main peak. To reinforce the validity of our measurements, we show in the second graph several XA spectra taken at various temperatures (not published yet). The splitting of the main structure is still clearly visible. Adding error bars is however a complicated task for the present experiment. The dark count of the detector is very low, i.e. low enough to not appear as fluctuations on our measurements. Other sources of possible uncertainties would be: - the variations of the liquid thickness which is controlled by the He pressure in the experimental chamber. -the variation of the photon flux. These two contributions are taken into account by dividing the detector signal by the corresponding curves (I0 and He pressure at each photon energy point). The reproducibility of the measurements is an indication that the error bars intensity is well below the intensity variation attributed to the splitting of the main peak, and a much better precision is obtained as compared to the results presented in reference 13.

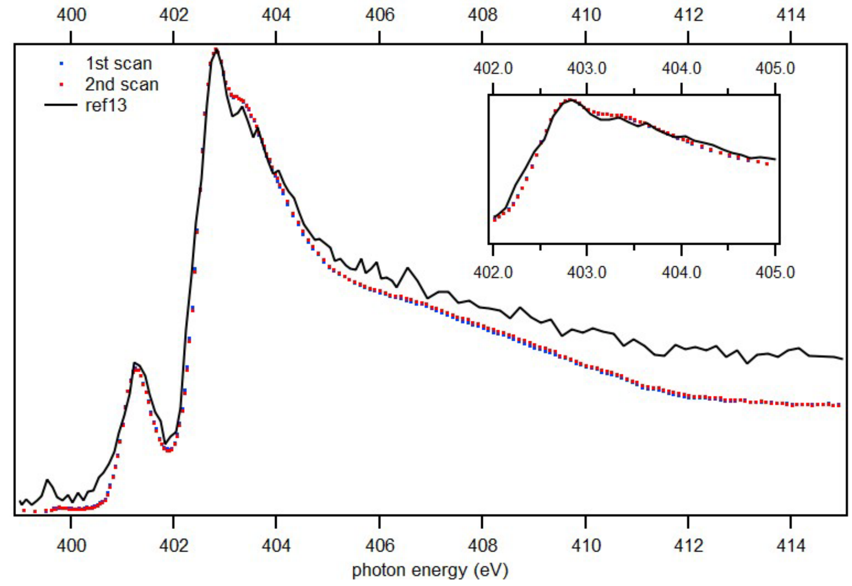

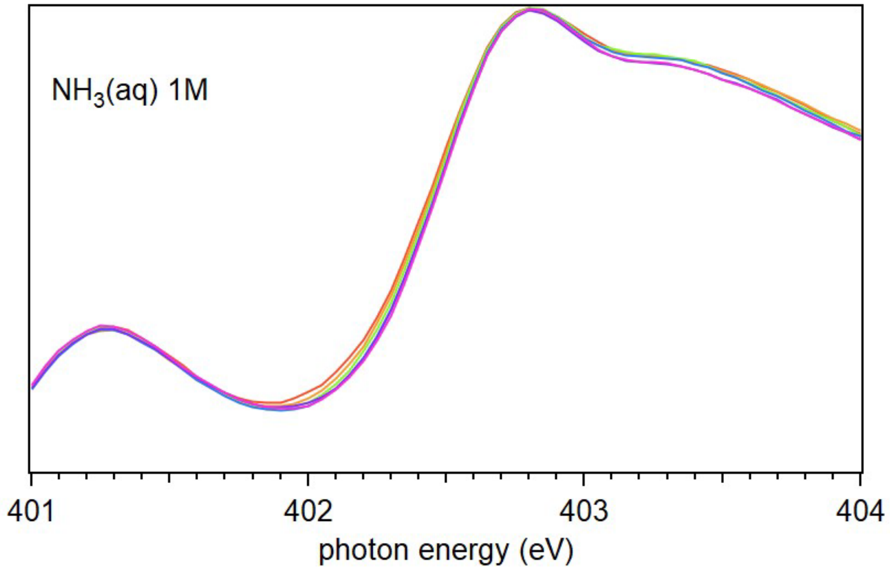

The error estimate should be also provided to the XFH calculations. Since the data are extracted from a limited number of points, statistical error could play a role. In this context, it should be also clarified whether the Gaussian kernel with a FWHM of 0.4 eV was used purely as a smoothing procedure or if there is a physical rationale behind it, such as accounting for the 1s core-hole lifetime. In that case, Lorentzian might be more appropriate yet this is a minor issue.

Authors' response: The std. error for the TP-DFT XFH spectrum has also been added to the XA spectrum presented in Figure S3, which is based on the sampling from the SCAN AIMD simulation, of comparable statistics to the BLYP-D3 AIMD simulation. The error bars are included in Figure S3 and not in Figure 1, to avoid cluttering the presentation.

Changes in revision: Standard errors were added to the computed TP-DFT spectrum in Figure S3. In addition a sentence was added "The precise choice of convolution does not have physical rationale, but it is likely smaller than the combined effect of lifetime broadening, vibrational broadening, and experimental broadening."

Some of the data (or closely related results) were already presented in Reference 13. This study introduces new measurements and hybrid MLCC3-in-HF calculations. However, the latter calculations fail to reproduce the observed splitting. The authors attribute this discrepancy to the lack of a certain type of orbital mixing in the hybrid CC3 calculations. Yet maybe, the difference has a more fundamental cause, as very different qualitative features are observed even for the C3 symmetry cluster model-most notably, the flipped intensity of the two peaks. The CC3 data should probably be considered a benchmark for the cluster case.

If so, an alternative hypothesis should be considered: perhaps the CC3 calculations are actually correct, and the lack of agreement with the experiment arises from a biased sampling of aqueous ammonia. This possibility is plausible given the generally poor performance of DFT functionals for liquid water.

Authors' response: We thank the reviewer for this important remark. Indeed, in general for spectrum simulations, and in particular for liquid solutions, there is always an uncertainty whether the lack of agreement with experimental data, is a consequence of limitations of the structural model or of approximations in the spectrum simulation. Likewise, agreement with experiment could be due to cancellation of errors from both sources.

We agree that for the $NH_3(H_2O)_3$ cluster, the CC3 calculations should be considered as a golden standard, and we argue for an essential semi-quantitative agreement of spectrum simulation using TP-DFT XFH and CC3 for the cluster. For the aqueous solution, however, the MLCC3-in-HF model is not necessarily capturing all effects in the PBC model. From our earlier work on QM/MM models in Ref. 26, we have tried to estimate the effects by evaluating different QM/MM models against the PBC model in TP-DFT spectrum simulations and we see that there are finite size effects with respect to the QM region. Hence, even though the MLCC3-in-HF model is more accurate than simple QM/MM models, we cannot due to computational cost fully test its limitations by including the full second solvation shell in the CC3 region.

From our study of the XA spectrum of liquid water in Ref. 29, we conclude that quantum dynamics may not alter the spectrum significantly. However, since the the choice of exchange-correlation functional in the AIMD simulations can influence the structure profoundly, we have also performed a AIMD simulations with the more recently developed strongly constrained and appropriately normed (SCAN) exchange-correlation functional, performing well for liquid water (Ref. 29 and references therein). This sampling of spectra was only computationally feasible for the TP-DFT XFH methods, but these spectra, as presented in a new figure in the SI, show a close resemblance with the sampling over the previous AIMD BLYP-D3 simulations. Hence, we have attempted to explore the limitation of the AIMD simulations and see that the main-edge splitting in the TP-DFT XFH spectrum simulations is a robust result.

Changes in revision: A sentence was added for the conclusion "We have explored two intrinsically different protocols for XA spectrum simulations, TP-DFT XFH and MLCC3-in-HF, sampled over the same set of configurations from an AIMD simulation. Although, we notice quantitative differences between the simulation protocols, where the splitting in the XA main-edge is only appearing in the sampled spectra for TP-DFT XFH and not for MLCC3-in-HF, we see qualitative similarities in the angular analysis supporting the proposed mechanism for the observed splitting. In addition, since the average XA spectrum sampled from the AIMD can be sensitive to the limitations of the approximations in DFT, we also sampled an AIMD simulation performed with the more recently developed strongly constrained and appropriately normed (SCAN) DFT functional [CITATION]. Due to the computational cost, this check was only performed on the TP-DFT XFH level and not on the

MLCC3-in-HF level. The details about the SCAN AIMD simulation are given in the SI together with the resulting XA spectrum in Figure S3, which shows an angular decomposition closely resembling that in Figure 4 and a weak dependence on the DFT approximation. "
Together with a description of the AIMD with the SCAN functional, a figure with the sampled XA spectra from the AIMD simulation was added to the SI mimicking Figure 4 in the manuscript.

This raises the question of why the C3 symmetry cluster was chosen as a model, assuming the ab initio dynamics data are reliable. Reference 13 suggests that such configurations are not typical for ammonia in liquid water - or is this not the case? The manuscript contains references to the connection between the model system and the ab initio dynamics data (e.g., lines 13-18 on page 7), but these explanations appear somewhat cryptic.

On the other hand, adopting a simple model with high symmetry might offer the advantage of applying group theory to rationalize the observed splitting and the trends.

Authors' response: The choice of using distortions from C_3 symmetry in an idealized model to analyze the XA spectrum of the aqueous ammonia solution was motivated by the trends seen in the angular analysis of the sampled spectra in Figure 4 and Figure 6. However, for the presentation, it was natural to establish how artificial distortions of the idealized cluster (Fig. 2-3) influences the XA spectrum, before discussing the spectral response to natural fluctuations in the solution (Fig.4,6).

Changes in revision: A sentence was added "The theoretical XA spectra in Figure 1 result from sampling over many different configurations with varying hydrogen bond distances and other fluctuations in coordination of ammonia and in inter-molecular geometries."

This study presents an interesting attempt to link molecular structure with X-ray absorption spectra of aqueous systems. It remains unclear whether this work fully capitalizes on the available data to establish a definitive relationship. While the connection between liquid structure and XAS seems clear, the precise nature of this relationship-and how best to model it-is not. I understand that additional data (e.g., measurements at different temperatures or using deuterated solutions) may not be available at this stage. Nevertheless, the interpretation should be presented with more caution.

Authors' response: We have evidence to support that we have identified a mechanism for splitting of the XA main-edge feature, but at the same time we have to admit that the situation is complicated with several contributions to the spectral shape of the nitrogen K-edge XA spectrum of aqueous ammonia. Hence, to reflect that we added comments to the conclusion and modified the abstract.

Changes in revision: A part of a sentence was added to the abstract "Although the spectral response to distortions is complex, we show that the".
Also a part of a sentence was added to the conclusions "There is a complex dependence of the XA spectrum on the local coordination and also more long-range interactions,"

jz-2024-03625m.R2

Name: Peer Review Information for "Symmetry Breaking around Aqueous Ammonia Revealed in Nitrogen K-edge X-ray Absorption"

Second Round of Reviewer Comments

Reviewer: 2

Comments to the Author

The authors have addressed all my questions. My main concern was the discrepancy between the experimental results, which show a small splitting, the TP-DFT method, which predicts a very pronounced splitting, and the high-level MLCC3-in-HF method, which shows no splitting. I greatly appreciate the new SCAN simulations, which demonstrate the (modest) sensitivity of the calculated outcome to different structural models. While this does not entirely rule out a structural cause for the discrepancy, it is certainly a valuable test.

However, I still find it puzzling why such large differences exist between the two computational approaches. I understand that performing CC3 calculations for two solvation layers is not feasible, but it should be possible to downgrade the TP-DFT calculations. If the authors use cluster models of increasing size, at what point does the splitting feature disappear?

My other concerns were more technical. The choice of the broadening parameter could perhaps be explained more clearly, but if an alternative choice would not change the interpretation of the splitting, further discussion may not be necessary.

Additionally, it would be useful to include the Cartesian coordinates of the cluster models in the ESI.

I would be pleased if the authors consider my suggestions above, as they could provide further insights. However, the manuscript is honestly written, and I have no reservations about its publication after minor revisions.

Author's Response to Peer Review Comments:

Response is given in Rebuttal letter

Second Revision: Manuscript ID jz-2024-03625m

To the editors of *The Journal of Physics Chemistry Letters*

Please find enclosed a revised version of the manuscript entitled
Symmetry Breaking around Aqueous Ammonia Revealed in Nitrogen K-edge X-ray Absorption by Michael Odelius, Sarai Dery Folkestad, Thanit Saisopa, Yuttakarn Rattanachai, Wutthigrai Sailuam, Hayato Yuzawa, Nobuhiro Kosugi, Alexander C. Paul, Henrik Koch, and Denis C´eolin.

We appreciate the possibility to make a minor revision in response to the comments of Reviewer 2 and modified the manuscript to clarify the issues raised by the reviewer. We would ask for this second revision to be considered for publication as an article in The Journal of Physics Chemistry Letters.

On behalf of the authors, sincerely Yours

Michael Odelius
Department of Physics
Stockholm University, Stockholm, Sweden
Stockholm March 20, 2025

Reviewers' comments

Reviewer: 2

Recommendation: This paper is publishable subject to minor revisions noted. Further review is not needed.

Comments: The authors have addressed all my questions. My main concern was the discrepancy between the experimental results, which show a small splitting, the TP-DFT method, which predicts a very pronounced splitting, and the high-level MLCC3-in-HF method, which shows no splitting. I greatly appreciate the new SCAN simulations, which demonstrate the (modest) sensitivity of the calculated outcome to different structural models. While this does not entirely rule out a structural cause for the discrepancy, it is certainly a valuable test.

Authors' response: We are grateful that Reviewer 2 recognizes the efforts and the value of the additional MD simulation with a different functional, although of course both the BLYP-D3 and SCAN exchange-correlation functionals are approximate.

However, I still find it puzzling why such large differences exist between the two computational approaches. I understand that performing CC3 calculations for two solvation layers is not feasible, but it should be possible to downgrade the TP-DFT calculations. If the authors use cluster models of increasing size, at what point does the splitting feature disappear?

Authors' response: We have not quite done what the reviewer proposes, but something very similar. Instead of simply increasing the cluster size, we have previously performed QM/MM calculations and varied the QM=DFT cluster size at the expense of the MM environment and we see in the red curves in Figure S11 in Ref. 26 how the mainedge changes in comparison to the fully periodic DFT calculation. The variations in the main-edge splitting and changes in the post-edge feature in the QM/MM models points to finite size effects with respect to the QM region. As the reviewer notes, the MLCC3-in-HF calculations are costly, and inclusion of another solvation shell in the correlated calculation is prohibitively expensive. Without such calculations, we cannot determine definitely if the lack of a main-edge splitting is due to an insufficient description of the environment beyond the first solvation shell. We note, however, that if a different convolution (broadening) scheme is chosen for the MLCC3-in-HF spectrum, a splitting (or shoulder) appears in the main-edge of the spectrum. In Figure S8 in Ref. 27, a splitting in the CCSD-inHF spectrum of approximately 0.5 eV appears when a Lorentzian broadening of 0.2 eV FWHM is applied. In the MLCC3-in-HF spectrum, a slightly narrower shoulder appears. With a Gaussian convolution of 0.4 eV FWHM (Figure S9 in Ref. 27), no splitting can be seen, since the peaks a sufficiently distinct to show up as two separate features. We added a few sentences discussing the splitting in the TP-DFT XFH and MLCC3-in-HF calculations.

Changes in revision: (In the beginning of the Results section:) We notice that there are indications of a splitting in the MLCC3-in-HF spectrum but only when the convolution scheme is less broad (See Figure S8 in Ref. 27) than for comparison to experimental data.
(In the end of the Results section:) The splitting of the main-edge at the TP-DFT XFH

2

level is overestimated (0.8 eV) in comparison to experiment (0.5 eV), whereas there are not sufficiently distinct peaks at the MLCC3-in-HF level to show any splitting. From previous quantum mechanics molecular mechanics (QM/MM) calculations varying the cluster size of the QM cluster at the expense of the surrounding MM region, we notice variations in the main-edge splitting and

changes in the post-edge feature in the QM/MM models pointing to finite size effects with respect to the QM region[26] (see red curves in Figure S11 in Ref. 26).

My other concerns were more technical. The choice of the broadening parameter could perhaps be explained more clearly, but if an alternative choice would not change the interpretation of the splitting, further discussion may not be necessary.

Additionally, it would be useful to include the Cartesian coordinates of the cluster models in the ESI. I would be pleased if the authors consider my suggestions above, as they could provide further insights. However, the manuscript is honestly written, and I have no reservations about its publication after minor revisions.

Authors' response: We very much appreciate the technical questions, and have described our pragmatic choice of broadening. The different convolution schemes in Figure S8 and Figure S9 in Ref. 27 show that the convolution scheme risk smearing the splitting, and also how the different CCSD-in-HF and MLCC3-in-HF levels of theory changes the splitting.

In the "Supporting Information Available", section we state that coordinates, some inputs and the spectra are available open access for download at https://doi.org/10.5281/ zenodo.14513943 (which is now finalized and published).
